# Discovery of functional non-coding conserved regions in the α-synuclein gene locus

**DOI:** 10.12688/f1000research.3281.2

**Published:** 2014-12-08

**Authors:** Lori Sterling, Michael Walter, Dennis Ting, Birgitt Schüle

**Affiliations:** 1 Parkinson's Institute and Clinical Center, Sunnyvale, CA 94085, USA; 2 Institute of Human Genetics, Eberhard-Karls-University Tübingen, Tübingen, 72076, Germany

## Abstract

Several single nucleotide polymorphisms (SNPs) and the Rep-1 microsatellite marker of the α-synuclein ( *SNCA*) gene have consistently been shown to be associated with Parkinson’s disease, but the functional relevance is unclear. Based on these findings we hypothesized that conserved cis-regulatory elements in the *SNCA *genomic region regulate expression of *SNCA*, and that SNPs in these regions could be functionally modulating the expression of *SNCA*, thus contributing to neuronal demise and predisposing to Parkinson’s disease.

In a pair-wise comparison of a 206kb genomic region encompassing the *SNCA *gene, we revealed 34 evolutionary conserved DNA sequences between human and mouse. All elements were cloned into reporter vectors and assessed for expression modulation in dual luciferase reporter assays.  We found that 12 out of 34 elements exhibited either an enhancement or reduction of the expression of the reporter gene. Three elements upstream of the *SNCA *gene displayed an approximately 1.5 fold (p<0.009) increase in expression. Of the intronic regions, three showed a 1.5 fold increase and two others indicated a 2 and 2.5 fold increase in expression (p<0.002). Three elements downstream of the *SNCA *gene showed 1.5 fold and 2.5 fold increase (p<0.0009). One element downstream of *SNCA *had a reduced expression of the reporter gene of 0.35 fold (p<0.0009) of normal activity.

Our results demonstrate that the *SNCA *gene contains cis-regulatory regions that might regulate the transcription and expression of *SNCA*. Further studies in disease-relevant tissue types will be important to understand the functional impact of regulatory regions and specific Parkinson’s disease-associated SNPs and its function in the disease process.

## Introduction

An emerging hypothesis is gaining increasing interest and is based on the concept that subtle overexpression of α-synuclein (α-syn) over many decades can either predispose or even cause the neurodegenerative changes that characterize Parkinson’s disease (PD). Neurons subjected to higher, non-physiological levels of α-syn might be more likely to be damaged by oligomerization or aggregation of this protein, eventually leading to the formation of α-synuclein-based neuropathological features of the disease ^1^.

It is now well established that both point mutations and large genomic multiplications of the α-syn ( *SNCA*) gene can cause an autosomal-dominant form of PD ^2– 10^. Furthermore, several association studies investigating genetic variants in the *SNCA* gene have found an increased risk for PD ^11– 19^. The finding that both qualitative and quantitative alterations in the *SNCA* gene are associated with the development of a parkinsonian phenotype indicates that amino acid substitutions as well as overexpression of wild-type α-syn are capable of triggering a clinicopathological process that is very similar to sporadic PD. Nevertheless, the precise mechanisms leading to α-syn-related pathology in sporadic PD in the absence of any α-syn mutations remain elusive.

The best characterized polymorphism in the *SNCA* gene is the Rep-1 mixed dinucleotide repeat which has been shown to act as a modulator of *SNCA* transcription ^14– 16^. The DNA binding protein and transcriptional regulator PARP-1 showed specific binding to *SNCA*-Rep1. These data were confirmed by a transgenic mouse model and demonstrated regulatory translational activity ^20^.

Functionally, *SNCA* expression levels in postmortem brains suggest that the Rep-1 allele and SNPs in the 3′ region of the *SNCA* gene have a significant effect on *SNCA* mRNA levels in the substantia nigra and the temporal cortex ^21^.

The promoter region of the *SNCA* gene has been recently examined in more detail in cancer cell lines and also in rat cortical neurons. Regulatory regions in intron 1 and the 5′ region of exon 1 have been shown to exhibit transcriptional activation ^22– 24^ as well as the NACP-Rep-1 region upstream of the *SNCA* gene ^14– 16, 20, 25^. Several transcription factors have been identified such as PARP-1 ^16^, GATA ^26^, ZIPRO1, and ZNF219 ^22^ to have an effect on regulating the *SNCA* promoter region.

There is mounting evidence that *SNCA* expression levels could be crucial for maintenance and survival of neurons and its misregulation could play a key role in the development of PD. Thus, the importance of thoroughly investigating the *SNCA* gene to fully understand its cis- and trans-acting elements and factors and for the functional interpretation of the PD-disease associated risk alleles is becoming increasingly clear.

The goal of this study was to investigate transcriptional regulation of the *SNCA* region using a complementary approach, under the hypothesis that conserved non-coding regions of the *SNCA* gene are comprised of transcriptional enhancers or silencers and thus modulate gene expression. This would mean that single nucleotide polymorphisms (SNPs) in these regions could influence the transcriptional pattern of the *SNCA* gene ^27^.

## Materials and methods

### Comparative genomics

Using comparative genomics, we searched for highly conserved non-coding sequences between human and mouse and identified 34 evolutionary conserved non-coding genomic regions (ncECRs) within the *SNCA* gene that are conserved between human and mouse.

We utilized two complementary browsers (Vista browser ( http://pipeline.lbl.gov/cgi-bin/gateway2) and ECR browser ( http://ecrbrowser.dcode.org/) to generate a conservation profile by aligning the human *SNCA* gene with its mouse counterpart in a pair-wise fashion. We applied established selection parameters for our search with >100bp in length and >75% identity ^28, 29^. In addition to the 111.4kb *SNCA* gene region, we included a 44.5kb upstream and a 50kb downstream intergenic region to also capture surrounding regulatory elements.

**Figure 1.**
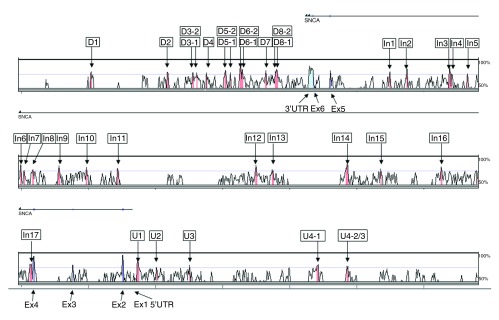
Vista plot from the *SNCA* region on chromosome 4q21. Panel shows human-mouse pair-wise comparison of Human genome May 2004 and Mouse Sept. 2005. Pink marked peaks represent ncECRs, turquoise marked peak represent the untranslated region (UTR) of *SNCA*, blue marked peaks represent exons. D1-D10 are conserved regions downstream of *SNCA*. In1-In17 are intragenic conserved regions, and U1-U4-2/3 are upstream of *SNCA*. The black arrow on top shows the transcription orientation.

We identified 34 ncECRs in the *SNCA* genomic region of 206kb on chromosome 4q21 (Chr.4: 90,961056-91,167082, UCSC Genome Browser on Human Mar. 2006 Assembly) by pair-wise comparison between human and mouse ( Figure 1). Ten of these DNA sequences were located downstream of the *SNCA* gene, 17 were intronic between exon 4 and 5, which is 92kb in length, and five were upstream of the *SNCA* gene ( Figure 1). None of the selected sequences overlapped with known expressed sequence tags (ESTs) or had an open reading frame of more than 20 amino acids in length, suggesting that these ncECRs are non-coding.

### Cloning and luciferase assays

To test, if the ncECRs exhibit enhancer or silencer activity, we cloned all identified regions in specific reporter vectors and measured their luciferase activity after transfection into neuroblastoma cells. For our studies, we used the pGL3 luciferase reporter vectors (Promega, Cat. No. E1751, E1741, E1771, E1761) and the human neuroblastoma cell line SK-N-SH. NcECRs identified through the comparative analysis ( Supplementary Table 1) were cloned upstream of a SV-40 promoter in the pGL3 promoter construct, transfected in SK-N-SH cells and assayed with the Dual-Luciferase ^®^ Reporter Assay System (Promega, Cat. No. E1910).

Some of these regions were combined in one vector because of their close proximity to each other. Primers with specific restriction sites (KpnI, BglII or XhoI from New England Biolabs Inc.) were designed to amplify the conserved elements, and PCR products with specific restriction sites were directly cloned into the pGL3 promoter vector to ensure correct orientation of the genomic elements ( Supplementary Table 1). All constructs were sequenced to ensure that no point mutations were introduced through the amplification and/or cloning process.

For transfection experiments, we used a 96-well format (Nunc, Cat. No. 167008). Cells were plated one day before transfection at a density of 3000–5000 cell/well to reach 90–95% confluency at the time of transfection, luciferase assays were performed 24hrs after transfection. SK-N-SH cells were maintained in Hyclone DMEM media (High Glucose, Fisher Scientific, Cat No. SH30081.02) with 10% Hyclone fetal bovine serum (Fisher Scientific, Cat No. SH30910.03) in 1× glutamine (Life Technologies, Cat. No. 25030-081) and 1× penicillin/streptomycin (Life Technologies, Cat. No. 15140-122). For SK-N-SH cells, we used 1:2 ratio of nucleic acid to transfection reagent (Lipofectamine ^®^ 2000 Transfection Reagent, Life Technologies, Cat No. 11668-019). For the luciferase assay, we used the Dual-Luciferase ^®^ Reporter (DLR™) Assay System (Promega, Cat. No. E1910) according to the manufacturer’s instructions in 96-well white plates, flat bottom (E&K Scientific, Cat. No. EK-25075). In this assay, activities of firefly and *Renilla* luciferases were measured sequentially in one sample. All assays were performed in quadruplicate and each experiment was repeated three times. Altogether, 12 data points were ascertained for each conserved region/construct.

### Statistical analysis

Differences among means were analyzed using two-samples student’s t-test. For differences in transcriptional activation of the luc+ gene, ncECRs were tested in quadruplicates in three independent experiments. Differences were considered statistically significant at p<0.05.

### Bioinformatic search for transcription factor binding sites (TFBS) with MatInspector (Genomatix)

To estimate the number of potential TFBSs and the number of interacting transcription factors (TFs) that could represent potential candidate proteins for our positive ncECRs, we used MatInspector in an *in silico* approach. We chose two elements for this bioinformatic analysis with MatInspector. The MatInspector software utilizes a large library of matrices for TFBSs to locate matching DNA sequences. The program assigns quality rating to matches and allows quality-based filtering and selection of matches. MatInspector can group similar or functionally related TFBSs into matrix families ^30^.

In addition to the original human-mouse comparison, we added the sequences for dog and cow for comparisons. Only the TFBSs were considered that were present in all four species, in the same orientation, and similar distance to each other. We ran two analyses with 10 and 15 nucleotides distance, respectively. We accepted only models in which at least four TFs can bind in a concerted way. Each TFBS can potentially bind several TFs.

We also computationally tested all possible TFs for interactions with the *SNCA* promoter region, which were retrieved from the proprietary ElDorado database (Genomatix, Munich, Germany). In this database, promoters are defined and ranked by transcription start sites, corresponding known mRNA or EST sequences and by orthologous conservation.

## Results

### Functional non-coding conserved elements within the *SNCA* genomic locus

**Figure 2.**
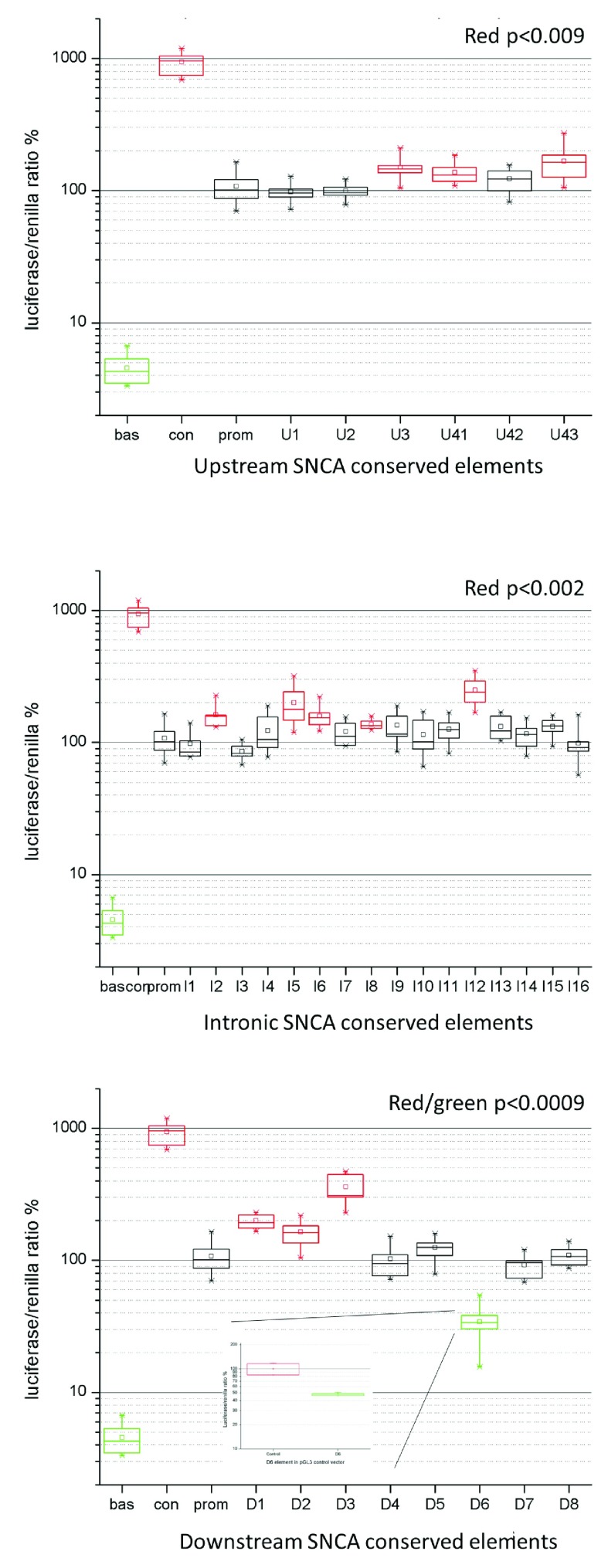
Non-coding conserved elements within the *SNCA* genomic locus show changes in luciferase assays. Panels **A–C** show the luciferase assay results of ncECRs upstream ( **A**), intragenic ( **B**), and downstream ( **C**) of the *SNCA* gene. The X-axis shows the ncECRs, the Y-axis shows the ratio of luciferase and renilla expression as percentage. Bas=pGL3 basic, Con=pGL3 control, prom=pGL3 promoter construct. All red or green box plot elements represent ncECRs that modulate expression significantly. The box plots show the median (horizontal line within box), the 25 and 75% tiles (horizontal borders of box), and the whiskers show the minimal and maximal values. Panel **C**, insert: Luciferase assay results of D6 element cloned into the pGL3 control vector construct.

Overall, 12 of 34 conserved non-coding elements exhibited either an increase or reduction of the expression of the luciferase reporter gene ( Figure 2 and Dataset 1). Three elements upstream of the *SNCA* gene (U3, U4-1, and U4-3) displayed a significant approximately 1.5 fold (p<0.009) increase in expression ( Figure 2A). Of the intronic regions, three showed a 1.5 fold increase (I2, I6, I8) and two others showed a 2 and 2.5 fold increase in expression (p<0.002), I5 and I12, respectively ( Figure 2B). Two elements downstream of the *SNCA* gene showed approximately 2 fold (D1 and D2) and 2.5 fold (D3) increase (p<0.0009) ( Figure 2C). One element D6 downstream of *SNCA* had a reduced expression of the reporter gene of 0.35 fold (p<0.0009) of normal activity ( Figure 2C, green) that was also confirmed after cloning the D6 element in a pGL3 control vector ( Figure 2C, insert). The pGL3 control vector contains the SV-40 promoter and a SV-40 enhancer element. The D6 element reduced the expression of the pGL3 control construct by ~50%, confirming that this element represents a repressor. Between 4 and 12 replicates were performed per ncECR.

These data provide experimental evidence that a significant proportion of the ncECRs show a regulatory function in the luciferase reporter assay.

### *In silico* analysis reveals potential binding of midbrain transcription factors to regulatory conserved regions

**Figure 3.**
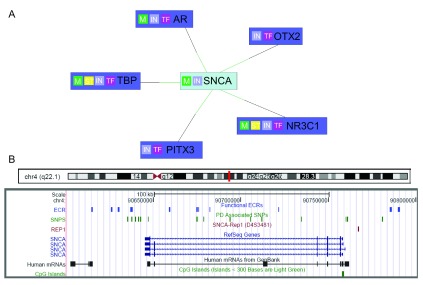
*In silico* analysis reveals midbrain transcription factors binding to two ncECRs. **A**. MatInspector network view of *SNCA* promoter interaction with TFs that also potentially bind to two ncECRs (I12 and D6) within the *SNCA* gene. M=gene product is part of metabolic pathway, IN=input gene, TF=transcription factor, ST=gene product is part of signal transduction pathway, green line=matches target promoter **B**. UCSC Genome browser custom track of PD associated SNPs (based on PD Gene metaanalysis), Rep1 allele and functional ECR regions on chromosome 4 (Human Genome Assembly Feb. 2009, GRCh37/hg19).

We performed MatInspector (Genomatix) analysis ^30^ on two elements (I12:chr4:90940532-90940786 and D6: chr4:90855871-90856339, Human Genome assembly NCBI36/hg18) with the highest fold change in the luciferase assay. In addition to the original human-mouse comparison to identify the ncECRs, we added the sequences from dog and cow. Only TFBSs that were present in all four species, in the same orientation, and similar distance to each other were considered. We ran two analyses with 10 and 15 nucleotides distance, respectively. We accepted only models in which at least four TFs can bind in a concerted way. Each TFBS can potentially bind several TFs. Interestingly, using this more restricted model, five factors showed an interaction with the *SNCA* promoter as well as with the ncECRs ( Figure 3A). These factors were the Paired-like homeodomain transcription factor 3 (PITX3), the Homolog of *Drosophila* orthodenticle 2 (OTX2), the Nuclear receptor subfamily 3, group c, member 1 (NR3C1) or glucocorticoid receptor (GCCR), the Androgen receptor (AR), and the general transcription initiation factor TATA box-binding protein (TBP).

It is intriguing to note that by searching for TFs that bind to both the promoter and the functional ncECR, several DNA-binding proteins were found that are linked to dopaminergic regulation and susceptibility for nigrostriatal impairment. Two of these TFs (PITX3 and OTX2) implicated in determination of a dopaminergic phenotype in the substantia nigra emerged from this preliminary search ^31, 32^. PITX3 has shown to be regulated in a negative feedback circuit through the microRNA mi-133b to fine-tune maintenance of dopaminergic neurons ^33^. In an association study, a SNP in the PITX3 promoter was reported to be associated with PD and might dysregulate expression of PITX3 ^34^ suggesting that transcription factors play a critical role not only in the development and differentiation of dopaminergic neurons, but also for cell maintenance and survival of dopaminergic neurons.

GCCR and AR belong to a class of nuclear receptors called activated class I steroid receptors. GCCR is a cytosolic ligand-activated transcription factor that regulates the expression of glucocorticoid-responsive genes. GCCR shows strong anti-inflammatory and immunosuppressive effects. Interestingly, impaired GCCR expression in a mouse model shows a dramatic increase in the vulnerability of the nigrostriatal dopaminergic neurons to a toxic insult of MPTP ^35^.

Taken together, this preliminary *in silico* screen resulted in very intriguing new candidates that might directly regulate *SNCA* expression and could play a role in the pathological processes that underlie PD.

Combined normalized raw datasets of Luciferase assays on *SNCA* conserved elements Data are ratios of luminometer readings for firefly luciferase and renilla luciferase. Ratios were normalized to Prom. Each non-coding element is labeled and data are presented under each element. Elements are organized according to Figure 2A–C.

## Discussion

A major focus in PD research has been on post-translational modification of α-syn. The alterations seen in PD that were linked to disease pathogenesis were nitrated α-syn and α-syn phosphorylated at serine 129 identified in Lewy bodies and Lewy neurites ^36, 37^, however, the gene transcription as a control point and its regulation in particular cell types or upon cellular signals has only been touched fairly recently in PD-relevant genes.

Our results show that potential regulatory regions are not restricted to the promoter of the *SNCA* gene as discussed in the introduction, but are likely to be located also in other intronic and intergenic regions ( Figure 3B). Comparing our results to similar screens, where conserved regions range from 8–45 elements ^38, 41^, we found a similar number of functional elements in our screen that show a high evolutionary conservation.

Not only the promoter region of a gene drives the transcription/expression of a gene. Also other cis-acting genomic regions within a certain gene, up to several hundred kb away, can serve as enhancers, silencers, or modifiers to ensure the accurate temporal and spatial expression of a gene by recruiting transcription activating or silencing factors that bind to them ^38^. There is ample precedence for this approach to analyze genomic regions of genes implicated in human disease. Mutations in those conserved elements were found to cause human genetic syndromes, for example SALL1/Townes-Brocks syndrome ^39^ or SHH/preaxialpolydactyly ^40^. Other groups have investigated the non-coding regulatory elements within disease genes such as RET (Ret proto-oncogene) and MECP2 (Methyl-CpG binding protein 2) and found multiple regulatory enhancer and silencer elements ^38, 41^.

### Transcriptional regulation of dopaminergic neurons

Computationally determining transcription factor binding sites is a challenging process and multiple prediction algorithms have been developed over the last decade (Cartharius 2005, Wu 2009, Mathelier 2013). Therefore our preliminary data should solely open the discussion and drive novel hypotheses for potential transcription factors that regulate transcription of the *SNCA* locus. Specific TFs seem to be directly involved in neurodegeneration and models of PD. TFs have been shown to be critical regulators for the development, maintenance and survival of dopaminergic neuronal populations ^42, 43^. E.g. forkhead transcription factor ( *Foxa2*) is responsible for early development of endoderm and midline structures. Foxa2 is specifically expressed in postmitotic dopaminergic neurons. Genetically engineered mice that are null for *Foxa2* are not viable, whereas heterozygotes for *Foxa2* develop major motor abnormalities starting at 18 months with an asymmetric posture, rigidity, and bradykinesia ^44^.

## Conclusion

This screen of evolutionary conserved genomic elements in the *SNCA* locus showed a number of functionally elements that in an *in vitro* assay modulated the expression of a reporter gene. Furthermore, we identified very intriguing new candidate transcription factors that could directly regulate *SNCA* expression and could, if binding is altered by genetic variants, play a role in the pathological processes that underlie PD. This is the first step to systematically analyze the *SNCA* locus to understand its transcriptional regulation in more detail. Further studies are needed in neuronal tissues (e.g. dopaminergic neurons derived from patient-specific induced pluripotent stem cells) to confirm these findings and expand the analysis to identify *SNCA*-regulating transcription factors. By defining the transcription factors that regulate expression and potentially overexpression of α-synuclein that can lead to neurodegeneration, we will be able to identify targets for novel therapeutic approaches for α-synucleinopathies including Parkinson’s disease.

## Data availability

*F1000Research*: Dataset 1. Combined normalized raw datasets of Luciferase assays on *SNCA* conserved elements, http://dx.doi.org/10.5256/f1000research.3281.d37452 ^45^

## Figures and Tables

**Supplementary Table 1.** Primer sequences and design for cloned ncECRs.

				HIndIII: CCC AAGCTT			
ECRs in *SNCA* locus on chromosome 4 Human Genome assembly NCBI36/hg18 (March 2006)				XhoI: CCGCTCGAG			
				KpnI: CGG GGTACC			
				BglII: GGA AGATCT			
							
ECR	Length	Identity	Location	Primers	PCR product length	Ann. Temp	Restriction sites within PCR product
D1	146bp	78.10%	chr4:90833665- 90833810	CGG **GGTACC**CACGAAATCGTGCCAAAAAT	601bp		no RE
				GGA **AGATCT**aagtcacaaggtcgaggcttt		60C	
D2	239bp	74.50%	chr4:90844830+ 90845413	CGG **GGTACC**tgcgaaattccacacaacat	584bp		no RE
				GGA **AGATCT**TCAGCAGATGGCATGGAATA		60C	
D3-1/2	143bp	72%	chr4:90848813- 90848955	CGG **GGTACC**AAGGGCTGACATTGGAATTG			no RE
	99bp	75.80%	chr4:90849405- 90849503	GGA **AGATCT**CCGCCTCTGAAAATAAGCAA	989bp	60C	
D4	110bp	73.60%	chr4:90850858- 90850967	CGG **GGTACC**GATGCAGCCATCAACTCTGA			no RE
				GGA **AGATCT**tgttggtagGCAGGAGAAATG	944bp	60C	
D5-1	241bp	75.90%	chr4:90853634- 90853874	CGGGGTACCACTTCCTTGGGTAGGCGAAT			BglII at 1143
D5-2	114bp	75.40%	chr4:90854429- 90854542	CCGCTCGAGGCTGAGATCACGCCACTGTA	1258bp	60C	use XhoI site
D6-1/2	234bp	83.30%	chr4:90855871- 90856104	GGA **AGATCT**CCATTCCCTCACCTCAAATG	582bp	60C	
	190bp	75.30%	chr4:90856150- 90856339	CGGGGTACCTCTGCATGAATGTGCAAACA			
D7	167bp	72.50%	chr4:90859690- 90859856	GGAAGATCTggggctgtagtgtggaaatc			no RE
				CGGGGTACCGGGCAGTGCATACTTGTCCT	856bp	60C	
D8-1/2	100bp	75%	chr4:90860722- 90860821	GGAAGATCTAGCTTCTGCCTTGTGTCTCC			no RE
	216bp	75.90%	chr4:90861289- 90861504	CGGGGTACCTTGAAGAACCCAAAATGCAA	1061bp	59C	
I1	192 bps	81.80%	chr4:90871989- 90872180	CCGCTCGAGaggataggctccaaccacct	840bp	60C	BglII at 571
				CGGGGTACCCAAATTCGGATCACGTAGGG			use XhoI site
I2	154bp	74%	chr4:90878220- 90878373	GGA **AGATCT**caggaattGGTGCAAAATCA	393bp	60C	
				CGG **GGTACC**aggggctgaccttcaagatt			
I3-1/2	276 bps,	77.50%	chr4:90887100- 90887375	GGAAGATCTtgaatgtgatggttcagcaaa	986bp	60C	no RE
	153 bps	76.50%	chr4:90887445- 90887597	CGGGGTACCgggaaggcaccctctaggta			
I4-1/2	194 bps	75.80%	chr4:90891860- 90892053	GGAAGATCTCCACCCCTCCACTTGACATA	899bp	60C	no RE
	100 bps	75.00%	chr4:90892381- 90892480	CGGGGTACCGCAATGGAACTGTGGTGATG			
I5-1/2	109 bps	76.10%	chr4:90893684- 90893792	GGAAGATCTCAGGCATGATTCCTCCCTTA	705bp	60C	no RE
	155 bps	73.50%	chr4:90893990- 90894144	CGGGGTACCCCATCAACATCCCAAGAACA			
I6	130 bps	74.60%	chr4:90894785- 90894914	GGAAGATCTccttgtgggtattcctgaacat	355bp	60C	no RE
				CGGGGTACCGAAGTTGCCTGAGCTCCAAT			
I7	187 bps	75.90%	chr4:90897558- 90897744	GGAAGATCTAGATGATGAGCAGGCAGTCC	432bp	60C	no RE
				CGGGGTACCcgaaccatagtggaaatcagg			
I8	112 bps	76.80%	chr4:90901290- 90901401	CCGCTCGAGaaggcttgattggacattgc	474bp	60C	BglII at 34
				CGGGGTACCctggaaagaattggccacaa			use XhoI site
I9	199 bps,	75.40%	chr4:90906237- 90906435	GGAAGATCTTGCAATGAAAACCACAATGG	561bp	60C	no RE
				CGGGGTACCtgtttatgttctgtattccaccaa			
I10	269 bps	74.30%	chr4:90926832- 90927100	GGAAGATCTtgggatgggtgggtaaatAG	899bp	60C	no RE
				CGGGGTACCtgtgtcaaggatGGGAAAAAG			
I11	108 bps	74.10%	chr4:90929480- 90929587	GGAAGATCTtcaaagcaaagatttttctcca	429bp	60C	no RE
				CGGGGTACCtggttccttttagcccaattt			
I12	255 bps	77.30%	chr4:90940532- 90940786	GGAAGATCTagggaagaggaaaagcttgg	669bp	60C	no RE
				CGGGGTACCAAGGTTGAAAAACCGTGGTG			
I13	127 bps,	75.60%	chr4:90945579- 90945705	CCGCTCGAGaggctctgggaccacaatta	578bp	60C	BglII at 328
				CGGGGTACCCCTCTTAACTTCTGGGCAACC			use XhoI site
I14	100 bps	75.00%	chr4:90958054- 90958153	GGAAGATCTtcccacctagaaccttacagga	701bp	60C	no RE
				CGGGGTACCACACTTGAGTGTTATGGACCCTCT			
I15	329 bps	76.30%	chr4:90961895- 90962223	GGAAGATCTttcaacgttgttgacacctca	490bp	60C	no RE
				CGGGGTACCccaGATAAATGCCATGCAAA			
I16	106 bps	75.50%	chr4:90976615- 90976720	GGAAGATCTCCCGTTACCACCTGTTGACT	651bp	60C	no RE
				CGGGGTACCgccattcgacgacaggttag			
U1	261 bps,	81.60%	chr4:90977921- 90978181	GGAAGATCTCCGTCCTCCTCCTCCTAGTC	883bp	60C	no RE
				CGGGGTACCATCACGCTGGATTTGTCTCC			
U2-1	105 bps,	76.20%	chr4:90980743- 90980847	GGAAGATCTTTCATGTTTTGTTTTCTCTTTGCT	860bp	59.5C	no RE
U2-2	100 bps,	75.00%	chr4:90981402- 90981501	CGGGGTACCcaccagagttgcagagttgc			
U3	329 bps,	73.90%	chr4:91004670- 91004998	CCGCTCGAGccatgcagttttccCCAATA	751bp	60C	BglII at 487
				CGGGGTACCTCTCTCTCATTTTTGGTTTTGACA			use XhoI site
U4-1			chr4:91,008,097- 91,008,809	GGAAGATCTCTGAAGTAGGGGGCTCTTCC	535bp	60C	no RE
				CGGGGTACCGAGTTCTTTGGCAGGAGTGC			
U4-2	131 bps,	74.00%	chr4:91009155- 91009285	GGAAGATCTtggagaattcagttgctattgg	837bp	60C	no RE
				CGGGGTACCTGTGTTGCCATAGTCACATGTTT			
U4-3			chr4:91,010,061- 91,010,758	GGAAGATCTAAGAAGAAGCAAGCCACACC	698bp	58C	no RE
				CGGGGTACCtttctgtagggtttatagtgtcca			
