# Supplementary material for: Discovery of functional non-coding conserved regions in the α-synuclein gene locus
Source: F1000Res. 2014 Dec 8;3:259. Originally published 2014 Oct 29. [Version 2] doi: 10.12688/f1000research.3281.2 (PMC4275022; doi:10.12688/f1000research.3281.2)
Supplement: Peer review history

# Peer review: Referee response for version 2

**DOI:** 10.5256/f1000research.6309.r6960

**Published:** 2014-12-09

**Authors:** Ornit Chiba-Falek

**Affiliations:** 1 Department of Neurology, Duke University, Durham, NC, USA

The authors replied adequately to my suggestions. I have no further comments.

I have read this submission. I believe that I have an appropriate level of expertise to confirm that it is of an acceptable scientific standard.

# Peer review: Referee response for version 1

**DOI:** 10.5256/f1000research.3521.r6590

**Published:** 2014-11-10

**Authors:** Jinglan Liu

**Affiliations:** 1 Clinical and Molecular Cytogenetics, St. Christopher's Hospital for Children, Philadelphia, PA, USA

The article by Sterling *et al.* has described the identification and functional analysis of evolutionally conserved non-coding elements that might be involved in the transcriptional regulation of the gene *SNCA*, mutations in which were associated with Parkinson’s disease. This is a very interesting, proof-of-concept article, with an attempt to provide pathogenic insight from the point of view of regulatory genomics for a complex human disease. I endorse the indexing of this manuscript.

It is now well recognized that ~98% of human genome do not code for proteins.  Comparative genomics studies revealed that the majority of evolutionally conserved regions consist of non-coding elements that that might be involved in regulating gene expression. Genome-wide association studies (GWAS) have showed that the majority (~93%) of SNPs contributing to human diseases or susceptibility lie outside protein-coding regions, and there are many non-coding SNPs have been demonstrated to be associated with common diseases and traits.

By identifying functionally significant non-coding elements for *SNCA*, Sterling *et al*.’s work

could lend a new perspective to study the genetic architecture of Parkinson’s disease, and promote further investigations on the pathogenic impact of non-coding elements and their regulatory networks on the clinical courses of Parkinson’s disease.

I have read this submission. I believe that I have an appropriate level of expertise to confirm that it is of an acceptable scientific standard.

# Peer review: Referee response for version 1

**DOI:** 10.5256/f1000research.3521.r6592

**Published:** 2014-11-06

**Authors:** Ornit Chiba-Falek, Lidia Tagliafierro

**Affiliations:** 1 Department of Neurology, Duke University, Durham, NC, USA; 2 Duke University, Durham, NC, USA

The paper by Dr. Schüle’s team describes on the identification of evolutionary conserved non-coding regions (ncECRs) in the α-synuclein (SNCA) gene and their assessment as candidate regulatory elements. The work coupled *in silico* and cell-based studies. By using a comparative genomic screen between human and mouse the authors identify 32 ncECRs, out of which 11 regions exert an effect on expression level using a luciferase reporter assay approach. Their findings add on previous reports in the field that have shown, using both luciferase reporter system and human brain tissues, that the SNCA gene contains cis-regulatory sites across the 3’ and the 5’ LD blocks that regulate its expression levels.

The study was well designed and thoroughly executed, the results are of interest to the scientific community of PD-genetics, and provide seeds for follow up studies. The paper is nicely written, logically flows and summarizes the literature in the field.  However, the authors should make major revisions according to the following comments:

1. There is some inconsistency regarding the number of the ncECRs identified in the initial screen between the different sections of the article (32, 34, 37). Please make the corrections where needed.
2. Additional necessary control for the Luciferase experiments is a pGL-(SV40) promoter vector harboring an insert of a scrambled sequence that its size range mimics  the average insert size of the tested ECRs. This is required to control for the ‘spacer’ effect of ECR lengths.
3. What method was used for the statistical analysis? It is also not clear in the text whether all significant changes were calculated in comparison to the SV-40 promoter-only vector. That should be described in details in the method section.
4. To demonstrate the important implication of this study the authors are recommended to follow up on an event as an example. That is to say, to evaluate the effect of a genetic variation, a PD-associated SNP, on the regulatory function of the corresponding ECR using the luciferase system established in this work. Figure 3 demonstrates overlap between PD associated SNPs and ncECR, connecting these dots will be of high significance.
5. Supp Table: there is a typo in the coordinates of D2. In the footnote include the human genome assembly of the coordinates.
6. Figure 2A X-axis: modify title to ‘upstream….’
7. Omit Figure 3A. Instead include a new panel to figure 3B that indicates the position of the putative binding sites of these TFs within SNCA locus.
8. The identification of Transcription Factor Binding Sites (TFBS) is an important step required in order to evaluate the transcriptional regulation network of the SNCA gene. To this end, the computational prediction of TFBS is a classic approach that gives preliminary data but should be interpreted with caution. Integration of the classic approach with new models described in Mathelier & Wasserman (2013) is highly recommended. The relation between TF motifs and *in vivo* binding sites is far from simple. The analysis lacks of information about the context of the identified sequences. TF are highly context-specific, and the same TF typically binds to different genomic binding sites in different conditions. Obtaining information about the context could be helpful in better understanding the possible involvement of the predicted sites as TFBS. While this is beyond the scope of this study, this topic should be thoroughly discussed in the discussion section.

We have read this submission. We believe that we have an appropriate level of expertise to confirm that it is of an acceptable scientific standard, however we have significant reservations, as outlined above.

# Response

**Published:** 2014-11-26

**Authors:** Birgitt Schuele

We very much appreciate the careful review and excellent comments, suggestions and future directions of the reviewers. We hope to have addressed all of the comments to the reviewers’ satisfaction.

There is some inconsistency regarding the number of the ncECRs identified in the initial screen between the different sections of the article (32, 34, 37). Please make the corrections where needed.

*Thanks so much for the comment. We made changes to reflect the correct number of 34 ncECRs. We combined counts for ncECRs that were located very closely in the luciferase assay to one ncECR therefore different numbers appeared in the text. That has been addressed.*

**

Additional necessary control for the Luciferase experiments is a pGL-(SV40) promoter vector harboring an insert of a scrambled sequence that its size range mimics the average insert size of the tested ECRs. This is required to control for the ‘spacer’ effect of ECR lengths.

*We have included in our analysis three controls: 1. The* *pGL3-Basic Vector which lacks eukaryotic promoter and enhancer sequences should not show any transcription activity. 2. The pGL3-Enhancer Vector contains an SV40 enhancer located downstream of luc+ and the poly(A) signal and is showing transcription a very high levels (enhancer element is 246bp in length). 3) pGL3-Promoter Vector contains an SV40 promoter upstream of the luciferase gene (promoter is 202bp in length).*

*Even though we have not directly included a control with scrambled sequence, we think that the ncECR elements that do not change transcription of* *luc* *+ provide enough evidence that the experimental system is valid. Of a total of 34 in silico determined elements, only 12 show an effect of transcriptional regulation. 22 elements did not change expression compared to* *pGL3-Promoter Vector.*

What method was used for the ***statistical analysis***? It is also not clear in the text whether all significant changes were calculated in comparison to the SV-40 promoter-only vector. That should be described in details in the method section.

*A description of the analysis of luciferase assays was lacking and has now been added as a paragraph at the end of Method section* *Cloning and luciferase assays and reads as follows:*

*“Statistical analysis:*

*Differences among means were analyzed using two-samples student’s t-test. For differences in transcriptional activation of the luc+ gene, ncECRs were tested in quadruplicates in three independent experiments. Differences were considered statistically significant at p<0.05.”*

To demonstrate the important implication of this study the authors are recommended to follow up on an event as an example. That is to say, to evaluate the effect of a genetic variation, a PD-associated SNP, on the regulatory function of the corresponding ECR using the luciferase system established in this work. Figure 3 demonstrates overlap between PD associated SNPs and ncECR, connecting these dots will be of high significance.

*This is an excellent suggestion and will definitely be conquered in future work with this system as this is the basis for the understanding of transcriptional regulation of the SNCA locus for potential translational applications. The presented study was intended to understand the basic changes in transcriptional regulation within the SNCA locus.*

**

Supp Table: there is a typo in the coordinates of D2. In the footnote include the human genome assembly of the coordinates.

*We corrected the coordinates for D2 which was a duplicate of D1 with the correct genomic location chr4:90844830+90845413 and added in the header the corresponding Human Genome assembly NCBI36/hg18 (March 2006).*

Figure 2A X-axis: modify title to ‘upstream….’

*Correction has been made. It reads now in Figure 2A “ Upstream SNCA conserved elements”. We also changed for consistency Figure 2B to “Intronic SNCA conserved elements” and capitalized Figure 2C “ Downstream SNCA conserved elements”.*

Omit Figure 3A. Instead include a new panel to figure 3B that indicates the position of the putative binding sites of these TFs within SNCA locus.

*We have modified Figure 3 according to the MatInspector network view with respective changes in the legend. We also included which genomic sequences have been analyzed in the text. Since this is a preliminary in silico analysis, we feel that the overview is sufficient and has to be validated in functional studies. As pointed out below by the reviewer, these analyses have to be taken with care and a grain of salt.*

**

The identification of Transcription Factor Binding Sites (TFBS) is an important step required in order to evaluate the transcriptional regulation network of the SNCA gene. To this end, the computational prediction of TFBS is a classic approach that gives preliminary data but should be interpreted with caution. Integration of the classic approach with new models described in Mathelier & Wasserman (2013) is highly recommended. The relation between TF motifs and *in vivo* binding sites is far from simple. The analysis lacks of information about the context of the identified sequences. TF are highly context-specific, and the same TF typically binds to different genomic binding sites in different conditions. Obtaining information about the context could be helpful in better understanding the possible involvement of the predicted sites as TFBS. While this is beyond the scope of this study, this topic should be thoroughly discussed in the discussion section.

*Thank you very much for this suggestion. Indeed, further studies are necessary to provide experimental evidence for the binding of predicted transcription factors. The analysis provided in this article was only a first step to model potential transcription factor binding sites and should stimulate further studies.*

*The reference Mathelier and Wasserman has been now included in the Discussion of the manuscript and reads as follows:*

*“Computationally determining transcription factor binding sites is a challenging process and multiple prediction algorithms have been developed over the last decade (Cartharius 2005, Wu 2009, Mathelier 2013). Therefore our preliminary data should solely open the discussion and drive novel hypotheses for potential transcription factors that regulate transcription of the SNCA locus.”*
